# Effects of an evidence service on health-system policy makers' use of research evidence: A protocol for a randomised controlled trial

**DOI:** 10.1186/1748-5908-6-51

**Published:** 2011-05-27

**Authors:** John N Lavis, Michael G Wilson, Jeremy M Grimshaw, R Brian Haynes, Steven Hanna, Parminder Raina, Russell Gruen, Mathieu Ouimet

**Affiliations:** 1 McMaster Health Forum, Hamilton, Canada; 2 Centre for Health Economics and Policy Analysis, McMaster University, Hamilton, Canada; 3 Department of Clinical Epidemiology and Biostatistics, McMaster University, Hamilton, Canada; 4 Department of Political Science, McMaster University, Hamilton, Canada; 5 Health Research Methodology Program, McMaster University, Hamilton, Canada; 6 Clinical Epidemiology Program, Ottawa Hospital Research Institute, Ottawa, Canada; 7 Department of Medicine, University of Ottawa, Ottawa, Canada; 8 Institute of Population Health, University of Ottawa, Ottawa, Canada; 9 Health Information Research Unit, McMaster University, Hamilton, Canada; 10 School of Rehabilitation Science, McMaster University, Hamilton, Canada; 11 CanChild Centre for Childhood Disability Research, McMaster University, Hamilton, Canada; 12 Evidence-based Practice Centre, McMaster University, Hamilton, Canada; 13 The National Trauma Research Institute, Alfred Hospital, Melbourne, Australia; 14 Departments of Surgery & Public Health, Monash University, Melbourne, Australia; 15 Department of Political Science, Université Laval, Québec, Canada; 16 Centre de Recherche du Centre Hospitalier Universitaire de Québec, Québec, Canada

## Abstract

**Background:**

Health-system policy makers need timely access to synthesised research evidence to inform the policy-making process. No efforts to address this need have been evaluated using an experimental quantitative design. We developed an evidence service that draws inputs from Health Systems Evidence, which is a database of policy-relevant systematic reviews. The reviews have been (a) categorised by topic and type of review; (b) coded by the last year searches for studies were conducted and by the countries in which included studies were conducted; (c) rated for quality; and (d) linked to available user-friendly summaries, scientific abstracts, and full-text reports. Our goal is to evaluate whether a "full-serve" evidence service increases the use of synthesized research evidence by policy analysts and advisors in the Ontario Ministry of Health and Long-Term Care (MOHLTC) as compared to a "self-serve" evidence service.

**Methods/design:**

We will conduct a two-arm randomized controlled trial (RCT), along with a follow-up qualitative process study in order to explore the findings in greater depth. For the RCT, all policy analysts and policy advisors (n = 168) in a single division of the MOHLTC will be invited to participate. Using a stratified randomized design, participants will be randomized to receive either the "full-serve" evidence service (database access, monthly e-mail alerts, and full-text article availability) or the "self-serve" evidence service (database access only). The trial duration will be ten months (two-month baseline period, six-month intervention period, and two month cross-over period). The primary outcome will be the mean number of site visits/month/user between baseline and the end of the intervention period. The secondary outcome will be participants' intention to use research evidence. For the qualitative study, 15 participants from each trial arm (n = 30) will be purposively sampled. One-on-one semi-structured interviews will be conducted by telephone on their views about and their experiences with the evidence service they received, how helpful it was in their work, why it was helpful (or not helpful), what aspects were most and least helpful and why, and recommendations for next steps.

**Discussion:**

To our knowledge, this will be the first RCT to evaluate the effects of an evidence service specifically designed to support health-system policy makers in finding and using research evidence.

**Trial registration:**

ClinicalTrials.gov: NCT01307228

## Background

Health-system policy makers make important decisions every day about the governance, financial, and delivery arrangements within which programs, services, and drugs are provided and about implementation strategies [1]. The nature of their decisions will vary according to the setting in which they work (*e.g.*, federal, provincial, or local government) and the role they play (*e.g.*, political staff, policy analyst, senior policy advisor, Assistant Deputy Minister, or elected official), among other factors. Systematic reviews are increasingly seen as a key source of information to inform these decisions [1]. Reduced bias and increased precision comprise the main advantages of systematic reviews that address questions about the effects of interventions [2]. Drawing on a systematic review that addresses *any *question constitutes a more efficient use of time for busy policy makers because the research literature has already been identified, selected, appraised, and synthesised in a systematic and transparent way. Additionally, a systematic review makes possible more constructive policy debates because stakeholders can focus on the synthesis and its local applicability rather than on which single study has greater credibility [3].

In order to make informed decisions, health-system policy makers need timely access to systematic reviews that can be easily retrieved using terminology that is understandable to them and that are presented in ways that facilitate rapid scanning for relevance, recency of searches for potentially relevant studies, the settings of studies included in the review, and quality of the review [3,4]. A systematic review of the factors that influence the use of research in policy making identified timing/timeliness as one of two factors that increased the prospects for research use among health-system policy makers [3,5]. However, when attempting to retrieve systematic reviews in a timely fashion, health-system policy makers typically cannot search all of the potential sources of systematic reviews. Moreover, policy makers typically cannot search most sources of systematic reviews, like The Cochrane Library, using terms with which they are familiar. The number and searchability of existing sources of systematic reviews become particularly frustrating when policy makers know there is likely to be a review available on a topical issue. Moreover, search results typically do not highlight the types of decision-relevant information that health-system policy makers are seeking [3,4].

One response to the similar types of issues faced by clinical decision makers has been the development of evidence services that provide regular email alerts about newly identified research products and a searchable database of these products[6]. However, no 'full-serve' evidence service currently exists to meet the needs of health-system policy makers. Existing evidence services that include health-system policy makers among their target audiences, such as E-watch (http://kuuc.chair.ulaval.ca/english/index.php) and CHAIN Canada (http://www.epoc.uottawa.ca/CHAINCanada/), do not focus on systematic reviews. Existing evidence services that focus on high-quality studies (not just systematic reviews), such as Evidence Updates (http://plus.mcmaster.ca/EvidenceUpdates/), do not target health-system policy makers[6].

To address this gap, we developed a full-serve evidence service for health-system policy makers. First, we developed Health Systems Evidence, which contains over 1,400 syntheses about governance, financial, and delivery arrangements within health systems and about implementation strategies relevant to health systems. By syntheses we mean both systematic reviews and two types of review-derived products, namely, policy briefs and overviews of systematic reviews [7]. A policy brief summarises how the findings from a number of systematic reviews pertain to a pressing problem, select options for addressing the problem, and key implementation considerations, whereas an overview provides a 'map' of all available systematic reviews on a broad health-system topic. The reviews have been (a) categorised by topic (*i.e.*, by health-system arrangement or implementation strategy), type of review (*i.e.*, policy brief, overview of reviews, Cochrane systematic review, systematic review, or systematic review protocol), and type of question addressed (*i.e.*, effectiveness, not effectiveness, and 'many'); (b) coded by the last year in which searches for studies were conducted and by the countries in which included studies were conducted; (c) rated for quality using the AMSTAR (A MeaSurement Tool for the 'Assessment of multiple systematic Reviews') instrument [8,9]; and (d) linked to available user-friendly summaries, scientific abstracts, and full-text reviews that are available free online [10].

Second, we identified systematic reviews in Health Systems Evidence that are neither available free online nor available through subscriptions held by the Ontario Ministry of Health and Long-Term Care (MOHLTC) and developed a mechanism to reimburse publishers for full-text downloads of these reviews.

Third, we developed the format for monthly email alerts, which (in tabular format) identifies new additions to Health Systems Evidence and describes the type of review, type of question addressed, health-system arrangement or implementation strategy addressed, and title of the review. A hypertext link for each review enables policy makers to view the availability of (and links to) user-friendly summaries, scientific abstracts, and the full-text review. A hypertext link to the online Health Systems Evidence webpage enables policy makers to view additional information about these same recent database additions, including the last year searched, quality rating, the countries in which included studies were conducted, and the complete citation. (Electronic newsletter width restrictions precluded having all fields presented in the monthly email alerts.)

Our goal is to evaluate whether (and how and why) a full-serve evidence service increases the use of synthesised research evidence by policy analysts and advisors in the MOHLTC as compared to a 'self-serve' evidence service. The full-serve evidence service comprises database access (an effort to facilitate policy makers' efforts to 'pull' in research when they need it), monthly email alerts about new additions to the database (a 'push' effort), and full-text article availability (an additional effort to facilitate pull). A systematic review found that simply providing information (in the form of clinical-practice guidelines) can change clinical behaviour,[11] which leaves us reasonably confident that we have the potential to achieve an increase in evidence use among health-system policy makers. Moreover, the results of a cluster randomised trial indicate that a full-serve evidence service increased practicing clinicians' utilisation of evidence-based information from a digital library [12].

## Methods/design

We will conduct this trial using a sequential explanatory mixed-methods design, [13] beginning with the randomised controlled trial (RCT) and then following up with a qualitative process study to explore the RCT findings in greater depth. For an initial two-month baseline period, all participants will receive the self-serve evidence service. For the following six-month period, the intervention group will receive the full-serve evidence service and the control group will continue to receive the self-serve evidence service. For a final two-month period, both groups will receive the full-serve evidence service. This protocol received ethics approval from the Hamilton Health Sciences/Faculty of Health Sciences Research Ethics Board at McMaster University (project number 10-267).

## RCT methods/design

### Study population and recruitment

To recruit participants who deal with health-systems issues on a regular basis, we will invite all policy analysts and policy advisors from one purposively selected division of the MOHLTC to participate in the RCT. All division staff members similarly face a relatively new expectation about obtaining training in finding and using research evidence (in the form of an indicator in annual performance reviews), as well as a new mandate for using the Ministry's 'Research Evidence Tool' for submissions that support decision making at the Ministry Management Committee and cabinet levels. Moreover, a trial endorsement letter will be signed by the Assistant Deputy Ministry responsible for this division. These contextual developments precede the launch of the trial and help to create a favourable climate for the use of research evidence among all potential trial participants.

Based on estimates provided to us in June 2009 by the MOHLTC, there are approximately 49 policy analysts (four are junior program and policy analysts) and 99 senior policy analysts in the division (n = 148). We do not yet have an accurate estimate of the number of policy advisors in the division; however, this group is likely to include roughly 20 people and all of them are likely to be senior policy advisors. By including all three levels of policy analysts and (if applicable) both levels of policy advisors, we will gather evidence from a diverse group that plays different roles in the policy-making process. For example, a policy analyst might conduct the initial, extensive 'workup' of an issue, whereas a senior policy advisor might write a short briefing note for the Minister.

Selecting this sample of policy analysts and advisors raises two applicability/generalisabilty issues. First, these RCT participants will differ in whether and when they received training on finding and using research evidence. Two of us (JNL and MGW) delivered a series of five one-day workshops for policy analysts and advisors at the MOHLTC between July 2008 and March 2009 (*i.e.*, 14 to 22 months before the trial will begin). We delivered five additional one-day workshops, one half-day workshop, and one half-day webinar for policy analysts and policy advisors, as well as one 1.5-hour workshop for more senior MOHLTC executives who set expectations for these staff, between January and March 2010 (*i.e.*, two to four months before the trial will begin). Given the division's expectation about training, we can assume that most RCT participants will have received the training. However, newly hired policy analysts and advisors may not have received the training, and others may not have been able to participate due to scheduling conflicts; those that have received the training will differ in the recency of the training. Second, these RCT participants will differ in their experience, which is somewhat related to their position (*i.e.*, level of policy analyst and level of policy advisor). To address each of these applicability/generalisability issues, we will stratify the randomisation based on past training and current position (see below).

### Intervention and control arms

We will conduct a two-arm RCT with a full-serve evidence service as the intervention arm and a self-serve version as the control arm. Participants allocated to the full-serve evidence service will receive the following:

• database (Health Systems Evidence) access (facilitating pull)

• monthly email alerts (push)

• full-text article availability (facilitating pull)

Participants allocated to the self-serve evidence service will receive only database access, which is already publicly available at http://www.healthsystemsevidence.org.

### Randomisation

Participants will be randomised using a stratified design. After completing the baseline questionnaire during the two-month baseline period, participants will be allocated to strata based on past workshop attendance (yes or no) and their position (policy analyst, senior policy analyst, or policy advisor). This two-layer stratification will produce six strata. Participants will be randomised after all those who consent to participate in the trial have completed the baseline questionnaire. We will assign a unique participant ID number to each participant and then provide the list of IDs to a biostatistician external to the research team who will conduct the randomisation and keep a log to provide a clear audit trail. The biostatistician will then communicate directly with a knowledge broker external to the research team who will be generating the email alerts and with the website server administrator at McMaster University who will be establishing which participants get access to which evidence service. The participants and investigators will be blinded to group assignment.

### Outcomes

Measuring the impact of knowledge transfer and exchange (KTE) interventions, such as the evidence service proposed here, poses significant challenges [14]. The ultimate goal of KTE interventions is typically to improve health. However, there is a long chain of potential causal relationships between an evidence service and improved health. For instance, the evidence service may influence the use of research evidence in different stages of the policy-making process, which in turn may influence decisions made by patients and healthcare providers (*e.g.*, healthcare professionals, teams, and institutions), which may in turn influence whether cost-effective programs, services, and drugs get to the patients who need them and have their desired impacts, and which in turn may translate into improved health [15]. Moreover, even the first relationship in this long chain is complicated by the competing influences on the policy-making process, such as institutional constraints within a political system, stakeholder pressure campaigns, values and beliefs held by key decision makers, and external factors such as the state of the economy [16-18]. Similar challenges arise when assessing the impact of KTE interventions, such as guideline-dissemination strategies, on clinical practice and on health [19-21].

Given these challenges, our primary and secondary outcomes for the trial are proxy measures for the use of research evidence in policy making. The primary outcome will be a measure of utilisation that is similar to the one used in a trial of the McMaster Premium Literature Updating Service (PLUS) [12]. Specifically, we will track the mean number of site visits/month/participant across trial groups during each period, that is, the baseline period, intervention period, and crossover period. We will also provide related descriptive measures such as the proportion of users per month in each of the full-serve and self-serve groups; the frequency with which the full monthly update page, systematic review records, and the more detailed documentation for each review (*e.g.*, user-friendly summaries, scientific abstracts, and full-text reports) are accessed; the mean number of minutes per month that participants use the database (with a 'time out' set at 60 minutes); and the number of times the monthly email alerts are forwarded.

Health Systems Evidence will be hosted on a secure server at McMaster University and will require a user login that will be used to accurately track their usage of the database. A user login is necessary because individuals from the MOHLTC do not have a consistent IP address when accessing external websites, which would preclude the collection of utilisation data if the site were hosted without requiring users to login. In addition, requiring user login will partially protect against contamination of the control group. However, we cannot rule out the possibility that individuals in the intervention arm of the study will forward monthly email alerts and full-text systematic reviews that are available only by subscription to individuals in the control arm; however, we will collect data about alert forwarding.

**Figure 1:**
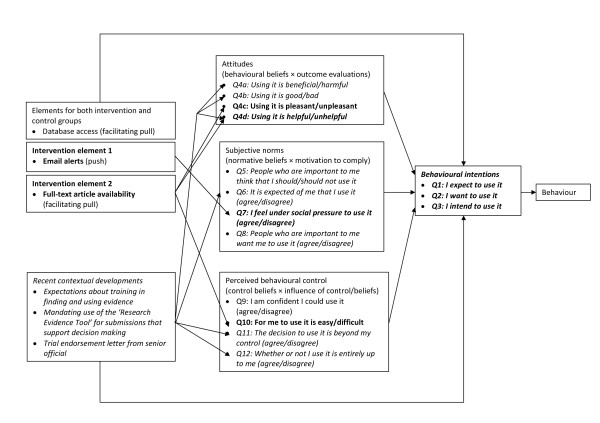
**Linkages among the intervention, contextual developments, and theory of planned behaviour constructs**.

For the secondary outcome, we will use a survey based upon the theory of planned behaviour to measure participants' intention to use research evidence. The theory of planned behaviour is a model of how human action is guided [22,23], and it consists of three variables--attitudes (*i.e.*, beliefs and judgments), subjective norms (*i.e.*, normative beliefs and judgments about those beliefs), and perceived behavioural control (*i.e.*, the perceived ability to enact the behaviour)--that shape the behaviour intentions of people, which is in turn a strong predictor of future behaviour [23-25]. In Figure 1, we outline linkages among the intervention, contextual developments (described above), and theory of planned behaviour constructs and measures.

The theory of planned behaviour has been extensively used and tested in the fields of psychology and healthcare. Systematic reviews conducted in the psychology field have demonstrated that the theory explains about 39% of the variance in intention and about 27% of the variance in behaviour [24,25]. A number of studies have demonstrated the feasibility of producing valid and reliable measures of key theory of planned behaviour constructs for use with healthcare professionals [26-28]. A systematic review suggests that the proportion of the variance in healthcare professionals' behaviour explained by intention was similar in magnitude to that found in the broader literature [29]. This successful transfer of the theory from individuals (as studied by psychologists) to healthcare professionals involved in an agency relationship with their patients (as studied by health-services researchers) bodes well for its further transfer to policy analysts and advisors involved in an agency relationship with Ministers and other senior officials.

Using a manual to support health researchers who want to construct measures based on the theory [23], we developed and sought preliminary feedback on a data collection instrument by first assessing face validity through interviews with key informants and then pilot testing it with 28 policy makers and researchers from 20 low-middle income countries who completed it after participating in a KTE intervention [30]. In addition, Boyko *et al. *(2010) found moderate test-retest reliability of the instrument using generalisability theory (*G *= 0.50) [31] when scores from a sample of 37 health-system policy makers, managers, professionals, citizens/consumers, and researchers participating in stakeholder dialogues convened by the McMaster Health Forum were generalised across a single administration, and even stronger reliability (*G *= 0.9) when scores were generalised across the average of two administrations of the tool [30]. In the reliability assessment by Boyko *et al. *(2010), the first administration of the tool immediately followed a McMaster Health Forum stakeholder dialogue, which may have promoted enthusiasm for using research evidence among participants. This likely produced higher measures of intention on the first administration of the tool as compared to the second, resulting in the lower *G *score. Given that we won't be administering the tool in a similar atmosphere of enthusiasm for using research evidence, we are confident in the level of reliability of the tool without two administrations at both baseline and follow-up. We modified the instrument by adding a question to measure the perceived usefulness of the intervention, as well as questions about participant characteristics.

We will administer the instrument during the baseline period, as well as at the end of the six-month intervention period, through a brief online survey that takes approximately 10 minutes to complete. We will use unique identifiers for each participant to ensure their responses to the previous survey are linked for calculations of before-and-after changes in their intention to use research evidence. We will follow up with participants who do not complete the survey once per week for three weeks to minimise the number of participants lost to follow-up.

### Data management and analysis

Data will be entered into SPSS 16.0 (IBM Corporation, Somers, NY) after all data collection has been completed. Analyses will be conducted by two members of the research team (SH and MGW), and during the analysis, neither they nor other study investigators will have access to the key linking the participants to their unique identifiers.

We will treat both outcome measures as continuous variables and analyse the change in these measures over time using a two-way mixed-effects linear repeated-measures analysis of variance (ANOVA), with the interaction of intervention by time as the main feature of interest. In addition, we will control for four variables--past workshop attendance, position (policy analyst, senior policy analyst, or senior policy advisor), branch within the division (of which there are six), and number of years working at the MOHLTC--using analysis of covariance. Given the likelihood that the distribution of the outcomes will be skewed, we will transform the data where necessary and possible, which may include adjusting the time period for which we calculate the mean number of site visits/participant (*e.g.*, calculating the mean over two months) if there are insufficient data for analysis. Moreover, as part of a secondary analysis, we will assess whether there is an interaction between each of these variables (entered as a fixed factor) and the outcome measures. We will also qualitatively compare the number of participants in the intervention and control groups that do not complete the follow-up survey and assess whether their baseline characteristics can help to explain their loss to follow-up.

For all analyses, we will use the intention-to-treat principle and report 95% confidence intervals; *p *values equal to or less than .05 (two-tailed) will be considered significant. For the primary outcome measure (mean number of site visits/month/participant), missing data are irrelevant because it is a naturalistic measure. For the secondary outcome measure (obtained through the survey), missing data can be taken into account through the use of a mixed-effects model.

### Statistical precision

Given a fixed sample size of at least 148 policy analysts and advisors in the division, a sample-size calculation is not relevant. Instead, we have calculated the level of statistical precision that we can expect given our fixed sample size. We had no mechanism to estimate the intraclass correlation coefficient (ICC) for measurements of the primary outcome for individuals over time. Therefore, we calculated estimates of statistical precision for ICCs of .2, .3, .5, .7, and .8 based on a six-month trial period with 80% power; an estimated standard deviation of 1.0; significance of .05; and 74 participants per study group (total n = 148, which does not include the as yet undefined number of senior policy advisors). Assuming the primary outcome data will be collected from all 148 participants at baseline and at six follow-up points (one per month), the time-averaged detectable difference (in standard deviation units) between the two groups is at best 0.27 (ICC = .2), which increases with successively greater ICCs to 0.30 (ICC = .3), 0.35 (ICC = .5), 0.40 (ICC = .7), and 0.42 (ICC = .8).

## Qualitative study methods/design

Given that this is the first RCT evaluating a KTE intervention for health-system policy makers (at least to our knowledge) and given the inherent limitations associated with measuring research use as an outcome, we will conduct a qualitative process study after the completion of the trial to explore the RCT findings in greater depth. The qualitative study will explore how and why the evidence service worked (or didn't work), including the role of past workshop attendance and position and the degree of contamination between the intervention and control groups.

### Sample

We will use a mixed-method sequential nested sampling procedure, whereby a larger sample is analysed in one study (RCT) and a subset of the larger sample is selected for further inquiry in the second study [32]. Specifically, 15 participants from each trial arm (n = 30) will be purposively sampled [33,34]. Our sampling criteria include RCT arm (*i.e.*, full-serve or self-serve evidence service), outcomes, past workshop attendance, position, branch within the division, and number of years working at the MOHLTC. We have assumed a 70% response rate (in keeping with our past experience with conducting qualitative studies involving health-system policy makers), which means that we should sample approximately 40 policy analysts and advisors in order to achieve a sample size of 30.

### Data collection

One-on-one semistructured interviews will be conducted either by telephone or in person (where possible) on participants' views about and experiences with the evidence service, including whether and how they used it (and the degree of 'contamination' between the two arms of the RCT, if any) and why, whether and how it was helpful in their work and why, what aspects were most and least helpful and why, and recommendations for next steps. Potential explanatory factors (for which we will probe) include past workshop attendance, position, branch within the division, and number of years working at the MOHLTC.

### Data management and analysis

We will tape and transcribe all interviews, use NVivo 8 (QSR International, Cambridge, MA) for data management, and use a constant comparative method for analysis [35-37]. Specifically, two reviewers will identify themes emerging from each successive wave of four to five interviews and iteratively refine the interview guide and emerging themes until we reach data saturation. This strategy will allow the reviewers to develop and refine codes and broader themes in NVivo 8 that reflect the emerging and increasing levels of nuance that result from the continuous checks that are involved in the constant comparative method [35,37]. The same reviewers will then apply the final analytic framework to all of the interview transcripts and conduct member checking once analysis is completed (*i.e.*, we will send a brief, structured summary of what we learned from the interviews and invite comment on it).

## Discussion

To our knowledge, this will be the first RCT to evaluate the effects of an evidence service specifically designed to support health-system policy makers in finding and using research evidence. While there have been a number of strategies developed to both support the production of policy-relevant research evidence and the identification and use of research evidence by health-system policy makers [1,38], rigorous evaluations of the effects of these strategies remains a critical gap in the KTE literature [38,39]. This study will begin to address this gap by providing a rigorous evaluation of the effects of a KTE intervention for policy makers and by examining how and why the intervention succeeds or fails. In addition, this trial will contribute to an emerging evidence base about similarities and differences in 'what works' in KTE across different target audiences [6,12,40].

The main potential limitation of the RCT is that it will be conducted within one division of the MOHLTC, and hence, there is the potential for contamination of study groups despite the use of a user-specific login. Given that many of the policy analysts and advisors work collaboratively, resources from the full-serve evidence service may be shared with those who had been allocated to the self-serve arm. Unfortunately, there is no mechanism to protect against this fully. However, we will adjust for variables (such as the branch in which the policy analyst is based) that may be correlated with degree of collaboration, and hence likelihood of contamination; we will measure the number of times that monthly email alerts are forwarded; and we will ask about contamination in the qualitative process study. Furthermore, if we find a significant amount of contamination through the qualitative study, it suggests that the full-serve evidence service is perceived as highly useful by those not allocated to receive it.

## Competing interests

Three of the authors (JNL, MGW, and JMG) were involved in the development, and remain involved in the continuous updating, of Health Systems Evidence, which is the intervention being tested in the trial.

## Authors' contributions

JNL conceived of the study, participated in its design, led its planning, and helped to draft the protocol. MGW participated in the design and planning of the study and drafted the protocol. JMG and RBH participated in the design of the study and provided feedback on drafts of the protocol. SH participated in the design of the study, supported the sample-size calculations, and provided feedback on drafts of the protocol. PR, RG, and MO provided feedback on drafts of the protocol. All authors read and approved the final manuscript.
